# Ten years of ear, nose and throat (ENT) services in Southern Africa: a scoping review

**DOI:** 10.1080/16549716.2024.2370102

**Published:** 2024-06-27

**Authors:** Lufunda Lukama, Colleen Aldous, Warren Kuhn, Charles Michelo, Chester Kalinda

**Affiliations:** a College of Health Sciences, Nelson R Mandela School of Clinical Medicine, University of KwaZulu-Natal, Durban, South Africa; b Department of Otorhinolaryngology, Head and Neck Surgery, Ndola Teaching Hospital, Ndola, Zambia; c Global Health Institute, Nkwazi Research University, Lusaka, Zambia; d Bill and Joyce Cummings Institute of Global Health, University of Global Health Equity, Kigali, Rwanda; e Howard College Campus, College of Health Sciences, School of Nursing and Public Health, University of KwaZulu-Natal, Durban, South Africa

**Keywords:** Ear, nose, and throat (ENT), health services, resource-limited setting, Southern Africa

## Abstract

**Background:**

While ear, nose, and throat (ENT) diseases are a substantial threat to global health, comprehensive reviews of ENT services in Southern Africa remain scarce.

**Objective:**

This scoping review provides a decade-long overview of ENT services in Southern Africa and identifies gaps in healthcare provision. From the current literature, we hope to provide evidence-based recommendations to mitigate the challenges faced by the resource-limited ENT service.

**Data Sources:**

PubMed, Web of Science, EBSCOhost, Cochrane Library, Cochrane Library, and Scopus.

**Review Methods:**

On several databases, we conducted a comprehensive literature search on both quantitative and qualitative studies on ENT services in Southern Africa, published between 1 January 2014 and 27 February 2024. The extracted data from the analyzed studies was summarized into themes.

**Results:**

Four themes in the fourteen studies included in the final analysis described the existing ENT services in Southern Africa: 1. Workforce scarcity and knowledge inadequacies, 2. Deficiencies in ENT infrastructure, equipment, and medication, 3. Inadequate ENT disease screening, management, and rehabilitation and 4. A lack of telehealth technology.

**Conclusion:**

The Southern African ENT health service faces many disease screening, treatment, and rehabilitation challenges, including critical shortages of workforce, equipment, and medication. These challenges, impeding patient access to ENT healthcare, could be effectively addressed by implementing deliberate policies to train a larger workforce, increase ENT funding for equipment and medication, promote telehealth, and reduce the patient cost of care.

## Paper Context

- **Main findings:** Ear, nose and throat (ENT) healthcare in Southern Africa faces critical shortages of workforce, equipment, and medication for disease screening, treatment and rehabilitation.
- **Added knowledge:** In this review, we identify challenges in the resource-limited Southern African ENT healthcare provision and provide evidence-based recommendations to mitigate these challenges.
- **Global health impact for policy and action:** Improving ENT service delivery in the resource-limited world requires deliberate policies that improve health worker training, expand financing and resource availability, incorporate new technology, and lower patient costs of care.

## Background

Diseases affecting the ear, nose, and throat (ENT) are a global phenomenon. With more than 1.5 billion people living with hearing loss, above 20 million afflicted with chronic otitis media, up to 40% suffering from allergic rhinitis, and 350,000 succumbing to head and neck cancer annually [1–4], ENT diseases have become an important public health concern. In resource-limited settings, where the impact of ENT diseases is greatest, ENT health care is characterized by severe shortages of human resources (ENT specialists, audiologists, and speech therapists), medication, and equipment for treatment [5–8]. In addition, funding for ENT healthcare, hearing and communication rehabilitation services are universally inadequate [9–11].

The impact of ENT diseases is substantial, with patients suffering diminished quality of life stemming from the resulting communication disabilities, mental ill-health, and missed career opportunities [12,13]. Furthermore, ENT diseases inflict considerable individual and health system financial strain, particularly in resource-limited countries [12,14].

Given the recent global push for countries to achieve Sustainable Development Goal (SDG) 3, which aims to ‘ensure healthy lives and promote well-being for all at all ages’ [15], addressing factors hindering the universal delivery of ENT healthcare has never been greater. Over the last decade, there has been a growing interest in improving ENT healthcare in Southern Africa, a region where the prevalence of extreme poverty exceeds 51% [16–18]. A 2017 survey of ENT services in sub-Saharan Africa reported a regional ratio of 1.2 million people per ENT surgeon, 0.8 million people per audiologist, and 1.3 million people per speech therapist, with scarce hearing testing and surgery [6]. The shortage of ENT healthcare workers (HCWs) is exacerbated by a lack of training opportunities, as well as limited access to higher technology and expensive healthcare resources [5,6,19]. Lukama et al. highlighted similar challenges in Zambia, documenting the four audiology booths, two flexible rhinolaryngoscopes, and four operating microscopes for the entire country, and critically scarce basic medication and surgical procedures for ENT [8,9]. In Mozambique, Malawi, Zimbabwe, and Zambia, several initiatives have been undertaken to address the poor access to ENT health care, including accelerating the training of ENT professionals and establishing more audiology units across the country [6,18,20].

While many ENT disease prevalence studies have been conducted in Southern Africa [21–24], there is as yet no review of the ENT service provision [25]. This scoping review aims to provide an overview of the otolaryngology services in Southern Africa over the past 10 years, categorize findings, and identify the gaps in healthcare provision. It further seeks to provide evidence-backed recommendations to mitigate the multifaceted challenges posed by ENT diseases in resource-limited settings.

## Methods

For this review, we were guided by Arksey and O’Malley’s 2005 scoping review framework [26] and followed the PRISMA 2020 updated guideline for reporting systematic reviews [27]. No human subjects were used, and therefore this scoping review did not require an Ethics Board review.

### Search strategy

We used the string ‘(ENT OR Otorhinolaryngology OR “ear, nose and throat” OR otolaryngology) AND (Southern Africa* OR Botswana* OR Lesotho* OR Eswatini* OR Swaziland* OR Namibia* OR South Africa* OR Angola* OR Malawi* OR Mozambique* OR Zambia* OR Zimbabwe* OR Comoros* OR Madagascar* OR Mauritius* OR “Democratic Republic of Congo*” or Seychelles* OR Tanzania*)’ to search the following databases for literature: PubMed, Web of Science, EBSCOhost, Cochrane Library, and Scopus. In our search, we considered all SADC countries, including Botswana, Eswatini (formerly Swaziland), Namibia, South Africa, Angola, Malawi, Mozambique, Zambia, Zimbabwe, Comoros, Madagascar, Mauritius, Democratic Republic of Congo, Seychelles, and Tanzania [28]. The dates initially searched were 1 January 2014, to 27 February 2024. On 16 May 2024, we updated our search to include literature published between 27 February 2024, and 16 May 2024. We retrieved all the literature, and a shared EndNote library (EndNote 21, Clarivate, Philadelphia, PA) was used for screening the data.

### Study selection

As we sought to limit our review to primary quantitative and qualitative research documenting the existence of ENT services and published in English, we excluded case reports and series, commentaries and editorials, disease epidemiological studies, validation studies, and those that investigated non-healthcare worker perceptions and opinions. Further, we excluded pilot studies, those trying new or low-cost technology, those investigating scoring systems, and descriptions of surgeries. We considered all studies in ENT, audiology, and speech language therapy (SLT).

At each subsequent database search, duplicates were removed with the title and abstracts simultaneously screened by three researchers (LL, WK, and CK). The full texts were assessed by five researchers (LL, WK, CK, CM, and CA), and the final full texts for inclusion were agreed on by all. Disputes were resolved by the first author (LL) before LL and CK independently extracted the data. Of the five researchers, two (LL and WK) were specialists in ENT, one (CK) in public health and research methods, one (CM) in epidemiology and biostatistics, and the last one (CA) in genetics and clinical and professional practice.

### Data extraction and analysis

The following data was captured onto a predesigned Microsoft Word sheet: author, year of publication, study design and methodology, setting, objectives, main findings, policy recommendations, and recommendations for future research. The data sheet was piloted on 5 (35.7%) of the included full texts, with modifications made to the sheet as necessary. All authors agreed on the data to be captured. The extracted data were verified by individuals who did not take part in the data extraction (WK, CA, and CM).

## Results

**Figure 1.**
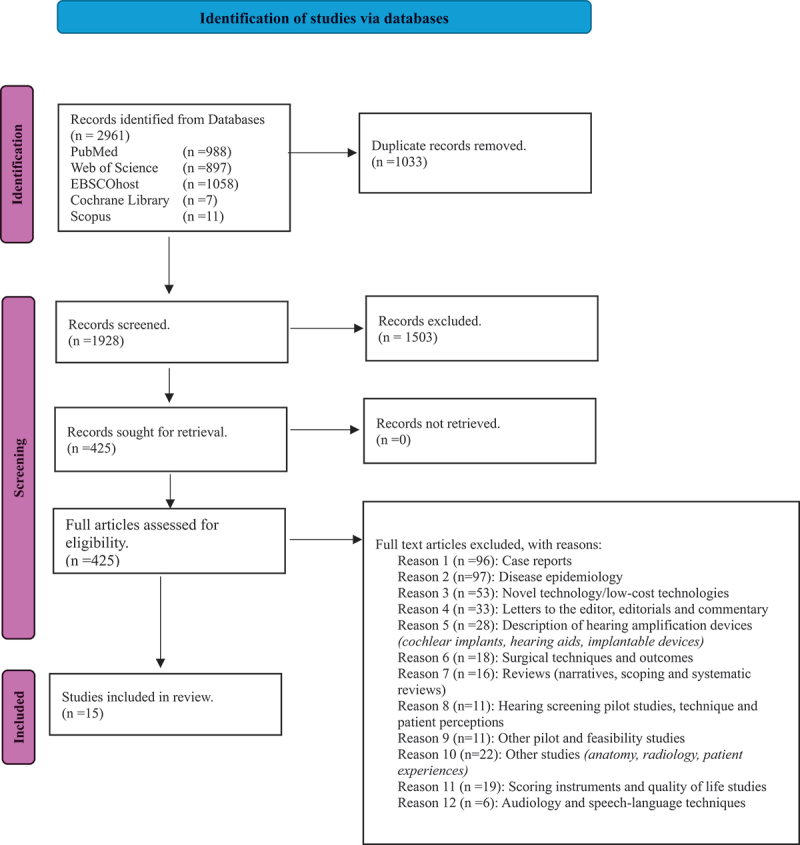
PRISMA diagram.

Our initial search retrieved 2961 articles. After removing duplicates, 1928 records underwent sequential title (559 included) and abstract screening, leaving 425 abstracts for full-text review. Applying the inclusion and exclusion criteria, we ultimately included 15 articles for thematic analysis, as shown in the PRISMA diagram in Figure 1. Of these, eight were cross-sectional surveys, one was a retrospective cross-sectional review of ENT patients’ clinical records, three were observational studies, and three employed mixed methods to gather data. A summary of the included articles is presented in Table 1.

**Table 1.** Summary of studies used in the review.

Author(s)/Year of Publication	Study title	Study design/Methodology	Study objectives	Main findings	Policy recommendations	Suggestions for future research
Bhamjee A, le Roux T, Schlemmer K, Graham MA, Mahomed-Asmail F. (2022)	Audiologists’ Perceptions of Hearing Healthcare Resources and Services in South Africa’s Public Healthcare System	Descriptive, telephonic survey of audiologists in public healthcare system hospitals across South Africa.	1. Describe audiologists’ perceptions of hearing healthcare resources and services within South Africa’s public healthcare system. 2. Investigate the gaps in available resources and services from a professional and clinical level perspective. 3. Provide data essential in gaining support for hearing loss from South Africa’s legislative sector and advocate for the integration of disability and quality of life concerns related to hearing loss on the national healthcare agenda.	1. 82% of audiologists perceived a lack of adequate hearing healthcare resources in South Africa’s public healthcare system. 2. Binaural amplification devices (invasive and non-invasive) for adults with bilateral hearing loss who adhered to the criteria for these devices were perceived to be unavailable in most hospitals. 3. Universal newborn hearing screening services, adult aural rehabilitation services, and follow-up care for all hearing devices post-warranty expiration were limited.	1. Increase financial budgets for procuring the necessary audiology equipment and hiring additional audiologists within the South African public sector hospitals. 2. Utilize the data obtained from the study to direct national policy on the improvement of hearing healthcare resources and service provision within South Africa, particularly within the public healthcare sector.	Focus on service evaluation studies to map hearing healthcare resources and services across various public sector hospital settings. It would be useful to relate the equipment data to patient pathways provided within services.
Moodley S. (2016)	Paediatric diagnostic audiology testing in South Africa	Retrospective observational study utilising a review of patient files in an early intervention program in Gauteng, KwaZulu-Natal and Western Cape Provinces of South Africa.	1. Determine the processes used for the diagnosis of paediatric hearing loss in South Africa, across both private and public healthcare sectors. 2. Profile the age of testing for each component of the diagnostic test battery.	1. Lack of equitable audiology practice and tests employed with paediatric clients across different regions and healthcare sectors in South Africa. 2. Each region was equally unlikely to complete a full, comprehensive diagnostic evaluation on paediatric clients. 3. Increased age of diagnosis of hearing loss	Use appropriate procedures and promote equity of services in paediatric diagnostic audiology by enhancing processes of diagnosis, audiological management, and intervention.	Conduct further studies on diagnostic practice and resources in South Africa and factors preventing adherence to international best practice guidelines for paediatric diagnostic audiology. Investigate the equitability of services across regions and healthcare sectors in South Africa, South Africa’s readiness to cope with the resources, personnel, and logistics necessary for accurate and timely diagnosis of pediatric hearing loss.
Lukama L, Kalinda C, Kuhn W, Aldous C. (2020)	Availability of ENT Surgical Procedures and Medication in Low-Income Nation Hospitals: Cause for Concern in Zambia	Descriptive cross-sectional survey of hospitals in Zambia.	Investigate ENT service provision in Zambia concerning the availability of surgical procedures and supply of essential drugs.	Limited availability of essential ENT surgical procedures, shortage of essential medication for ENT conditions, and low percentage of hospitals with an ENT budget.	Effectively collaborate with high-income countries to enhance ENT health, decentralize health services and foster government-led deliberate policy to increase ENT funding as urgent requirements for healthcare improvement.	Periodically audit the availability of surgical procedures and supply of essential drugs concerning ENT service provision in Zambia.
Mulwafu W, Nyirenda TE, Fagan JJ, Bem C, Mlumbe K, Chitule J. (2014)	Initiating and developing clinical services, training and research in a low resource setting: the Malawi ENT experience	Mixed methods, analysing data predating and following the establishment of ENT services in Malawi.	To reflect on the new Malawian ENT experience and to propose guidelines to poorly resourced countries.	Establishment of ENT services by the first Malawian ENT specialist, the implementation of training programs for clinical officers, and the significant burden of ENT conditions in Malawi (15,284 consultations of which 2.7% had surgery and 2.1% had foreign body removals in 2012)	1. Locally train more specialists and mid-level cadres, develop infrastructure, and promote visiting specialists for capacity building. 2. Improve funding for ENT healthcare and leverage the locally established health delivery models.	Research to develop a master plan to include types and numbers of professionals, infrastructure, funding, and relevant data collection to fine-tune the program and training.
Kanji A, Jamal A. (2023)	Roles and reported practices of paediatricians in the early identification and monitoring of hearing impairment in high-risk newborns and infants	Cross-sectional, descriptive online survey of paediatricians in the South African private and public healthcare sector.	Identify the role and reported practices of paediatricians in the early identification and monitoring of hearing impairment in high-risk newborns and infants.	1. Paediatricians showed variability in early identification protocols and referral practices for audiological evaluation 2. Despite the variability, paediatricians generally had sufficient knowledge regarding the assessment of hearing impairment and their role in early hearing detection and intervention programs. While 98% believed that they formed part of the multidisciplinary team, only 69% reported that they had actually been part of it.	Ensure standardization and consistency in paediatrician’s approach to early identification protocols and referral practices for client audiological evaluation.	Future research should explore the effectiveness of information sharing by audiologists and investigate the impact of information sharing on the early identification and monitoring of hearing impairment.
Fagan JJ, Otiti J, Aswani J, Konney A, Diom ES, Baidoo K, et al. (2019)	African head and neck fellowships: A model for a sustainable impact on head and neck cancer care in developing countries.	Observational study via emailing of questionnaires to past fellows to gather data.	1. Determine the effectiveness of the African clinical fellowship model in establishing and growing head and neck services in Africa. 2. Identify barriers to establishing head and neck services in underserved African countries, explore ways to improve the fellowships, identify the barriers to replicating such fellowships in other African countries, and assess whether such fellowships are a good model for developing countries.	1. Seven fellows established new multidisciplinary cancer teams. 2. Head and neck operations had increased by >335%, as had the complexity of the surgery. 3. There was effective transfer of surgical skills to trainees. All considered head and neck fellowships to be the best model to grow head and neck care.	1. Establish and strengthen training institutions in developing countries for a sustainable service. 2. Specialist training should be appropriate for developing world practice. 3. Individuals and institutions in developed countries to make targeted interventions by providing training and support.	Future research could focus on evaluating the long-term impact of such fellowships on surgical training programs and specialized surgical services in developing countries, as well as exploring additional strategies to improve access to surgery in these regions.
Canick J, Petrucci B, Patterson R, Saunders J, Htoo Thaw M, Omosule I, et al. (2023)	An analysis of the inclusion of ear and hearing care in national health policies, strategies and plans	Observational study involving a literature search for specific applicable keywords.	1. Characterize the inclusion, and explore the representation of ear and hearing care (EHC) in national health policies, strategies, and plans (NHPSPs) 2. Identify the under-prioritization of EHC in NHPSPs, especially in low- and middle-income countries.	1. The 100 included documents mentioned EHC keywords significantly less than comparison terms, with mention of hearing in 15 documents, ears in 11 documents and deafness in 3 documents. There was a mention of HIV/AIDS in 92 documents, tuberculosis in 88 documents and malaria in 70 documents. 2. Documents in low- and middle-income countries included significantly fewer mentions of EHC terms than those of high-income countries.	1. Analyse barriers to EHC policy development through stakeholder involvement and qualitative research on political priority, as has been done in global surgery, oral health and other fields. 2. Utilize tools developed by the WHO to facilitate the prioritization of EHC by national governments.	Future research must be designed with key stakeholders including policymakers, funders, civil society organizations and research experts in mind, to address knowledge gaps in EHC.
Khoza-Shangase K, Kanji A, Ismail F. (2020)	What are the current practices employed by audiologists in early hearing detection and intervention in the South African healthcare context?	Cross-sectional quantitative online survey completed by audiologists and dually qualified speech therapists and audiologists.	Explore the current reported practices of audiologists in early hearing detection and intervention (EHDI) in South Africa, specifically focusing on newborn and infant hearing screening services in healthcare institutions throughout the country.	1.83.7% of audiologists and speech therapists were involved in newborn hearing screening (NHS), with over half adopting the targeted screening approach instead of universal NHS services (UNHS). 2. Over 60% of audiologists reported that NHS is and should only be conducted by audiologists; with minimal evidence of task shifting found. 3. There was no standardised screening protocol, with challenges around budget allocation for EHDI revealed.	1. Increase resource allocation for AHDI and maintain functional assisted devices to ensure uninterrupted use of hearing aids, and improve strategic planning for effective translation of knowledge into practice. 2. Review the feasibility and applicability of EHDI guidelines released by the Health Professions Council of South Africa and address shortages hindering the EHDI process. 3. Adopt standardised protocols to allow for efficacious EHDI implementation as well as the ability to evaluate programme success and/or failure.	Future research should aim for a higher response rate to better reflect the overall number of qualified audiologists’ speech-language therapists and audiologists, especially from the public healthcare sector to allow for better generalization of the findings and address the limitations of the survey.
Petrucci B, Okerosi S, Patterson RH, Hobday SB, Salano V, Waterworth CJ, et al. (2023)	The Global Otolaryngology-Head and Neck Surgery Workforce	Cross-sectional electronic survey using e-mail outreach conducted with the REDCap platform.	Establish comprehensive workforce metrics for global otolaryngology-head and neck surgery (OHNS) with updated data and quantitatively monitor the surgical workforce to drive focused capacity-building and innovation in care delivery.	1. The global OHNS clinician density was 2.19 OHNS clinicians per 100,000 population. 2. Regionally, Europe had the highest clinician density (5.70 clinicians per 100,000 population) whereas Africa (0.18 clinicians per 100,000 population) and Southeast Asia (1.12 clinicians per 100,000 population) had the lowest. 3. More than 70% of countries/territories reported that the OHNS workforce provided at least 50% of surgical care for ear, nose and throat conditions. 4. 20% reported the absence of any OHNS training programs. The Western Pacific and African Regions had the lowest proportion of countries with OHNS training programs.	Quantitatively monitor the surgical workforce, urgently invest in training programs, promote collaboration among institutions or countries to create regional educational programs, encourage task sharing to address the workforce shortfall, and enhance financing to support infrastructure and multidisciplinary services.	Future research to focus on developing detailed, country-specific workforce characterizations, determining subspecialty OHNS workforce and access to subspecialty training. Further, it should asses data with other metrics to establish benchmarks for the minimum OHNS clinician density, and characterizing the accessibility and quality of OHNS training programs to guide resource allocation for workforce expansion.
Lukama L, Aldous C, Michelo C, Kalinda C. (2023)	Ear, Nose and Throat (ENT) disease diagnostic error in low-resource health care: Observations from a hospital-based cross-sectional study	Retrospective cross-sectional review of ENT patients’ clinical records at a resource-limited tertiary hospital.	The study objectives are to determine the diagnostic accuracy of non-ENT clinicians, estimate the appropriateness of referrals for ENT specialist care, and assess the prevalence of diagnostic error among non-ENT trained health workers in low-resource settings.	Non-ENT clinicians misdiagnosed 67.4% and inappropriately referred 50.4% for ENT specialist care. Compared to those aged 0–5 years, patients aged 51–87 years were 1.77 (95%CI: 1.03–3.04) fold more likely to have a referral misdiagnosis for specialist care. Agreement in diagnosis between the ENT specialist and nonENT clinicians was poor (κ = 0.0001).	Prioritize and invest in more effective and accelerated ENT training for clinicians in low-resource settings to improve diagnostic accuracy and reduce diagnostic errors and inappropriate patient referrals. This could involve developing and implementing training programs specifically focused on ENT diseases for non-ENT clinicians, as well as potentially establishing international partnerships to train specialists locally. Enhance ENT telemedicine in Zambia to improve patient’s access to quality healthcare	Evaluate the effectiveness of accelerated ENT training for clinicians in Zambia to improve diagnostic accuracy and reduce diagnostic errors and inappropriate patient referrals
Lukama L, Kalinda C, Aldous C. (2019)	Africa’s challenged ENT services: highlighting challenges in Zambia	Cross-sectional descriptive survey on hospital medical managers.	Document the profile of hospitals offering ENT services in Zambia and examine the ENT service with regard to human resources, infrastructure, and availability of equipment in hospitals.	1. ENT services in Zambia were poor at all levels of hospital care, with critical shortages in human resources, infrastructure and equipment. 2. Only 2.9% of hospitals had an audiology booth and 1.6% had a speech therapy room. Of the second and third level hospitals, 9.1% had flexible rhinolaryngoscopes, 18.2% had operating microscopes and 68.2% had adenotonsillectomy sets. 3. Zambia had 4 ENT surgeons, 1 Audiologist and no Speech Therapists.	1. Engage more developed countries for collaborative training programs, boost political will to adequately finance ENT health systems, prepare national plans to address hearing loss, and implement strategies outlined in the National Health Strategic Plan 2017 to 2021. 2. Establish audiology units at all secondary and tertiary hospitals.	1. Research to evaluate the effectiveness of collaborative efforts between the Zambian government and partnering organizations, and strategies laid out by the government of Zambia to reduce morbidity associated with ENT diseases. 2. Conduct an assessment of the impact of national plans to address hearing loss.
Bhamjee A, le Roux T, Schlemmer K, Graham MA, Mahomed-Asmail F. (2022)	Perceptions of Telehealth Services for Hearing Loss in South Africa’s Public Healthcare System	Cross-sectional electronic survey and focus group to gather information on audiologists’ perceptions of telehealth services.	Describe audiologists’ perceptions regarding telehealth services for hearing loss within South Africa’s public healthcare system, understand the potential of telehealth to make hearing care more accessible, and support guidance for future implementation of telehealth services.	1. Audiologists recognized the potential of telehealth services to improve hearing healthcare within the public sector, and most (84.1%) were willing to utilize it. 2. The use of telehealth services increased during the COVID-19 pandemic (from 7.2% to 19.6%), highlighting its importance in the continuity of hearing healthcare services during physical distancing. 3. Prominent barriers to telehealth primarily included the unavailability of equipment for remote assessments, internet-related barriers, and limited IT infrastructure.	1. Use of data from the study to guide policy toward the improvement of hearing healthcare resources in South Africa, especially within the public healthcare system. 2. Improve public sector hospital access to IT support and internet, equipment, infrastructure, and human resources to enhance telehealth services for better accessibility to audiological services.	Exploration of in-person data collection methods in future research, and focus on audiologists familiar with telehealth services to determine its value to patients within South African public sector hospitals.
Wylie K, McAllister L, Davidson B, Marshall J. (2016)	Communication rehabilitation in sub-Saharan Africa: A workforce profile of speech and language therapists	Mixed methods survey on Speech and Language Therapists (SLTs) within Anglophone countries of sub-Saharan Africa (excluding South Africa).	Exploration of the workforce providing rehabilitation services to people with communication disabilities (PWCD) and to describe the characteristics, education, experience, and stability of a sample of SLTs in sub-Saharan Africa.	1.59% of the African SLTs had received their first (entry-level) speech and language therapy qualification inside Africa. 2. 45% of African SLTs had less than 2 years of experience in practice, while 73% of non-Africans had between 2- and 10 years of experience. 3. African nationality respondents reported a strong intention to stay,	Ensure SLT workforce stability.	Explore the motivation and experiences of SLTs in SSA, as well as the perspectives of African stakeholders on the contributions and stability offered by foreign SLTs. Identify professional support needs for SLTs entering the workforce to ensure their work is of high quality and relevant to the culture and context. Further explore the relevance of the SLT profession in the region, including how it fits with existing rehabilitation models and how practices derived from a European belief framework can evolve to meet the needs of the African populations.
Mulwafu W, Ensink R, Kuper H, Fagan J. (2017)	Survey of ENT services in sub-Saharan Africa: little progress between 2009 and 2015	A questionnaire-based cross-sectional study on ENT surgeons and audiologists in sub-Saharan Africa.	Determine the status of ear, nose, and throat (ENT), audiology, and speech therapy services in sub-Saharan Africa.	1. Over 6 years, the number of ENT surgeons, audiologists, and speech therapists per 100,000 people had declined in four countries, and there remained a severe shortfall in these cadres when compared to the UK. 2. Unavailability of basic equipment, lack of ENT training facilities and audiological rehabilitation, low awareness of the burden of ENT pathology, as well as poor human resources management limited the provision of ENT services.	1. Invest in infrastructure and equipment and create training centres for ENT specialists, audiologists, and speech therapists. 2. Promote collaboration with countries or organisations in high-income settings for the advancement of ENT health. 3. Train more mid-level cadres.	Future research to include more countries for improved accuracy.
Pillay M, Tiwari R, Kathard H, Chikte U. (2020)	Sustainable workforce: South African Audiologists and Speech Therapists	A descriptive, retrospective study of South Africa’s Audiology and Speech Therapy workforce.	Examine the demographic profile and the supply, need and shortfall of Audiologists and Speech Therapists in South Africa.	1. Most (47.4%) of the professionals were registered as both Audiologists and Speech Therapists, 33.5% as Speech Therapists and 19.1% as Audiologists. 2. Around 88.5% of Audiologists and Speech Therapists were practising independently, most (42.6%) in the Gauteng province. 3. The professionals were mainly classified as white (59.7%). 4. In 2017, in best guess scenario, there was a supply–need gap of around 2800 professionals. Uncorrected, this shortfall would remain same by the year 2030.	1. Train more mid-level healthcare providers to provide core services for many underserved populations at primary health centres like PHC clinics, given the critical shortage of qualified providers. 2. Develop clear staffing norms (possible benchmarks) toward ensuring equity in human resources for health distribution of Audiologists and Speech Therapists.	Explore issues surrounding the current framework regulating training of Audiologists, Speech Therapists, and associated professionals, in order to respond adequately to future requirements.

Of the 15 included studies, three were from Zambia, six from South Africa, one from Malawi, three regional (sub-Saharan Africa and Africa), and two global. Four themes emerged from the 15 articles: 1. Scarcity of the ENT workforce and rehabilitation services, and knowledge inadequacies; 2. deficiencies in infrastructure, equipment, and medication; 3. inadequate ENT disease screening and management; and 4. telehealth and innovation.

### Scarcity of the ENT workforce and rehabilitation services, and knowledge inadequacies

In the included studies, workforce shortages, inadequate ENT knowledge among clinicians, and disparities in clinician density across income groups contributing to challenges in accessing ENT services were prominent findings [5–7,9,11,17,29,30]. The ENT clinician density in Southern Africa was significantly lower (0.18 clinicians per 100,000 population) than the global average of 2.19 clinicians per 100,000 population [7], particularly in Zambia and Malawi, where ENT specialists, audiologists, and speech and language therapists were critically short [9,11,31]. These few clinicians are largely confined to urban centers, leaving rural areas underserved [9,31]. The scarcity of the ENT workforce is compounded by severe deficiencies in healthcare workers’ knowledge of basic ENT disease management, further compromising disease outcomes [29]. Despite the majority of speech and language therapists (SLTs) having attained a minimum of a bachelor’s degree in SLT [30], most other health workers involved in ENT health in countries like Zambia and Malawi were mid-level cadres without undergraduate degrees [9,11]. These mid-level cadres, including Clinical Officers (Diploma holders in Clinical Medical Sciences or equivalent) and Medical Licentiates (holders of an Advanced Diploma in General Medicine, a Specialty of Medicine, or a Bachelor of Science in Clinical Science), often manage patients at primary care and secondary facilities [9]. However, these health workers have limited knowledge of ENT disease management due to inadequate training [29].

### Deficiencies in infrastructure, equipment, and medication

Several studies in Southern Africa described inadequate infrastructure and widespread unavailability of equipment, medication, and surgical procedures sufficient for ENT health care [8–11,32,33]. Hearing amplification devices like hearing aids were scarce [32]. They emphasized the urgent need for policy changes to improve ENT healthcare, including increased budget allocations and making essential medication more available. In addition to the scarce human resources, equipment, and funding for ENT healthcare, both hearing and communication rehabilitation services were largely unavailable in Southern Africa, notably in Zambia, Malawi, and South Africa [9–11]. Public health facilities were more affected than private facilities [33].

### Inadequate screening, management, and rehabilitation of disease

ENT disease screening, management and rehabilitation services were largely unavailable [5,5,6,8–10,17,30,32–36]. Ear and hearing conditions were severely underrepresented in national health policy in Southern Africa [35], with several studies documenting the disparities in the practice of audiology, scarce testing facilities, and inadequate resources for hearing screening across different regions and healthcare sectors [10,32,33]. Universal newborn hearing screening services, adult rehabilitation services, and follow-up care for hearing were limited in Southern Africa [5,9,32]. Owing to the shortage of audiologists in the region, newborn hearing screening in many places is done by pediatricians, underscoring the pivotal role pediatricians play in the early identification and referral of hearing impairment [36]. However, even in places where most neonatal hearing screening is conducted by audiologists, there remains a lack of standardized screening procedures [33].

The scarcity of ENT treatment services was a universal phenomenon [6,8,9,30,34]. Hearing rehabilitative surgery like tympanoplasty, middle ear prosthesis, bone-anchored hearing aids, and cochlear implants are largely unavailable [5]. To make these services more available, fellowships in Africa have been effective in increasing the provision of specialized surgical services and establishing multidisciplinary teams, addressing the shortage of specialists in the region [17].

### Telehealth

While telehealth has enormous potential to improve the efficiency of hearing healthcare in the public sector, it is largely unavailable due to a lack of resources, equipment, and infrastructure [37]. Despite the overwhelming enthusiasm by audiologists to use telehealth technology, its uptake was very low [37].

## Discussion

In this review, we described the ENT service in Southern Africa, uncovering the gaps in the service to inform policy. The review underscores critical deficiencies in the ENT workforce, medication and surgery for ENT healthcare, and screening and rehabilitation services, particularly in Zambia and Malawi. These deficiencies perpetuate the high morbidity and mortality among patients with ENT diseases and poor quality of life, retarding progress toward achieving the third SDG and promoting universal well-being for all [12,13,15]. With most of the studies in our scoping review documenting case reports and series (22.9%) and the epidemiology of disease (22.4%), the dearth of literature on the availability and quality of ENT services in many Southern African countries calls for renewed effort into research that focuses on patient access to health care [32].

### Workforce limitations

The Southern African ENT clinician density of 0.18 per 100,000 population found in this scoping review is much lower than those in other low-resource regions, including Central America (0.68 per 100,000) and Southeast Asia (1.12 per 100,000) [38]. With 70% of lower-middle, and 100% of low-income countries having less than one ENT clinician per 100,000 population [38], this shortage of ENT specialists, audiologists, and SLTs noted is concerning, leading to disparities in access to care and compromised disease outcomes. While targeted training programs for mid-level ENT cadres and specialists have been implemented in South Africa [17,39], Malawi [18], and Zambia [9] over the last decade, little progress has been made in improving the number of available ENT healthcare workers [5,6]. To strengthen workforce capacity, a deliberate policy to increase training facilities should be strengthened, as noted in the Cape Town Head and Neck Fellowship [17]. This policy should include increased funding for ENT training in existing schools, mobilization of specialised ENT workforce for teaching, and offering retention incentives for teaching faculty. Further, public-private partnerships for ENT training must be prioritized to assure sustainability of these training programs [17]. Besides training specialists, we encourage health systems to provide shorter training programs for mid-level cadres as an urgent measure to strengthen the primary health care system and pathways to care. The mid-level cadres perform diagnostic and treatment functions that are traditionally thought of as the responsibility of doctors, usually in primary and secondary healthcare settings. They include clinical officers, health officers, medical assistants, medical licentiates, clinical associates, and others who are trained to diagnose and manage common medical, maternal, and child health (MCH) and surgical conditions [40]. These mid-level cadres are impactful in the provision of accurate screening and diagnostic ENT services, promoting task-shifting and sharing for a more accessible ENT service [18,41].

Improving ENT healthcare worker competence through carefully planned, targeted short courses as continuous medical education in resource-limited settings is essential to empowering healthcare workers to deliver quality care within their scope of practice [42,43]. Coupled with telehealth, patients can achieve faster access to specialist care than the standard healthcare pathway [44,45]. The underutilization of telehealth technology found in this review presents a missed opportunity to improve accessibility to ENT services, especially hearing health. As a strategy to increase the adoption of telehealth, investments in infrastructure, equipment, and training are crucial [37,45]. Healthcare systems should prioritize the integration of telehealth platforms into existing healthcare systems, providing technical support and capacity-building initiatives.

### Infrastructure, medication, and equipment deficiencies

The inadequate infrastructure and lack of essential equipment and medication for ENT care are significant barriers to service delivery [8,29]. Policy reforms aimed at increasing budget allocations for ENT healthcare, prioritizing the procurement of essential equipment and medication, and enhancing infrastructure development are critical to improving access to ENT healthcare. Additionally, leveraging public-private and international partnerships to improve resource mobilization and distribution could expedite the acquisition of critical resources, as has been done in Mozambique [20], Zambia, Malawi, and Zimbabwe [6,9]. In addition to resource mobilization, partnerships should be used for the transfer of skills to local clinicians to achieve a sustainable service [46,47].

The scarcity of ENT disease screening, management, and rehabilitation services for ENT diseases calls for the prioritization of ENT initiatives in national health policy [35]. Healthcare providers must advocate for the establishment of universal newborn hearing screening programs and expanding adult rehabilitation services to meet the United Nations’ third Sustainable Development Goal (good health and well-being for all ages) [48].

### Financial barriers to access to ENT services

Financial barriers significantly impede access to ENT services. With widespread poverty in Southern African countries, many people lack financial resources for healthcare, including diagnostic tests, medication, and procedures [49]. The limited resources in healthcare facilities perpetuate the economic barriers to healthcare [50], and despite healthcare ostensibly being free for low-cost services, patients still bear a substantial out-of-pocket (OOP) expenditure on healthcare [51]. While there is still a dearth of information about patient out-of-pocket expenses for ENT diseases in Southern Africa, a number of studies have looked into OOP costs for various medical and surgical conditions. The average patient out-of-pocket expenditure in rural Malawi is USD 2.72, while the median out-of-pocket expenditure on surgical disease in South Africa is over R100, which is reportedly high given the country’s current economic circumstances [52,53]. Therefore, reducing patient OOP expenditures must be an integral part of efforts to improve access to ENT health services. For example, the Zambian government introduced the National Health Insurance Scheme (NHIS) in 2019 to serve as a pooled resource for affordable healthcare [54], which significantly reduced patient OOP expenditure for those enrolled. Other African countries, including Rwanda, Botswana, Tanzania, and Nigeria, have utilized the concept of community health insurance to reduce patient OPP expenditure for better access to health care [55]. However, the national coverage of these public health insurance schemes remains small [55], and governments must scale up efforts to make these insurance schemes more available to citizens.

The results of this scoping review of Southern Africa are consistent with our personal experiences in Zambia and South Africa, with critical shortages of a trained workforce, medication, equipment, and rehabilitation services. Therefore, the proposed evidence-based strategies to address the constraints encountered by under-resourced ENT services are critical to improving ENT healthcare in Southern Africa.

### Limitations of the study

We acknowledge our limitations in this scoping review, including our exclusion of literature published in languages other than English (1 study), the subjective nature of screening for inclusion of articles, and the strictness of our inclusion criteria, which may have excluded relevant literature for ENT healthcare. Further, our search terms may not have captured all the relevant literature, but we reduced this risk by performing trial literature searches in the considered databases to ensure relevant literature was captured. Our inclusion of five researchers in the screening process also reduced the impact of subjectivity in the literature review.

Despite our comprehensive search encompassing 15 countries – including three studies from Zambia, six from South Africa, one from Malawi, three regional (sub-Saharan Africa and Africa), and two global – only a few studies were retrieved for analysis. The paucity of studies on ENT services in the remaining Southern African countries may be due to the undocumented nature of these services, as suggested by the included studies. Consequently, our results may underestimate the extent of the challenges facing ENT services in these regions. A multi-national audit of ENT services using healthcare workers would overcome this limitation.

## Conclusion

The scoping review highlights critical deficiencies in ENT services in Southern Africa, including the scarcity of a workforce, medication and equipment for healthcare, inadequate infrastructure, and gaps in screening and rehabilitation services. Deliberate policies to enhance health worker training, increase funding and resource availability, integrate telehealth technology into healthcare, and reduce the patient cost of care are imperative to improving ENT service delivery.

## Data Availability

The data used to write this review is included in the manuscript.
